# Bilingualism and Cognitive Reserve: A Critical Overview and a Plea for Methodological Innovations

**DOI:** 10.3389/fnagi.2015.00249

**Published:** 2016-01-12

**Authors:** Noelia Calvo, Adolfo M. García, Laura Manoiloff, Agustín Ibáñez

**Affiliations:** ^1^School of Philosophy, Humanities and Arts, Institute of Philosophy, National University of San JuanSan Juan, Argentina; ^2^Cognitive Psychology of Language and Psycholinguistics Research Group, Laboratory of Cognitive Psychology, CIPSI (CIECS-CONICET), National University of CórdobaCórdoba, Argentina; ^3^Laboratory of Experimental Psychology and Neuroscience, Institute of Cognitive Neurology, Favaloro UniversityBuenos Aires, Argentina; ^4^National Scientific and Technical Research CouncilBuenos Aires, Argentina; ^5^Faculty of Elementary and Special Education, National University of CuyoMendoza, Argentina; ^6^UDP-INECO Foundation Core on Neuroscience, Diego Portales UniversitySantiago, Chile; ^7^Universidad Autónoma del CaribeBarranquilla, Colombia; ^8^Centre of Excellence in Cognition and its Disorders, Australian Research CouncilSydney, NSW, Australia

**Keywords:** bilingualism, Alzheimer's disease, cognitive reserve, retrospective studies, experimental research

## Abstract

The decline of cognitive skills throughout healthy or pathological aging can be slowed down by experiences which foster cognitive reserve (CR). Recently, some studies on Alzheimer's disease have suggested that CR may be enhanced by life-long bilingualism. However, the evidence is inconsistent and largely based on retrospective approaches featuring several methodological weaknesses. Some studies demonstrated at least 4 years of delay in dementia symptoms, while others did not find such an effect. Moreover, various methodological aspects vary from study to study. The present paper addresses contradictory findings, identifies possible lurking variables, and outlines methodological alternatives thereof. First, we characterize possible confounding factors that may have influenced extant results. Our focus is on the criteria to establish bilingualism, differences in sample design, the instruments used to examine cognitive skills, and the role of variables known to modulate life-long cognition. Second, we propose that these limitations could be largely circumvented through experimental approaches. Proficiency in the non-native language can be successfully assessed by combining subjective and objective measures; confounding variables which have been distinctively associated with certain bilingual groups (e.g., alcoholism, sleep disorders) can be targeted through relevant instruments; and cognitive status might be better tapped via robust cognitive screenings and executive batteries. Moreover, future research should incorporate tasks yielding predictable patterns of contrastive performance between bilinguals and monolinguals. Crucially, these include instruments which reveal bilingual disadvantages in vocabulary, null effects in working memory, and advantages in inhibitory control and other executive functions. Finally, paradigms tapping proactive interference (which assess the disruptive effect of long-term memory on newly learned information) could also offer useful data, since this phenomenon seems to be better managed by bilinguals and it becomes conspicuous in early stages of dementia. Such considerations may shed light not just on the relationship between bilingualism and CR, but also on more general mechanisms of cognitive compensation.

## Introduction

Our daily activities have profound cognitive consequences. In particular, they may influence our chances to develop Alzheimer's disease (AD)—the most prevalent form of dementia (Ferri et al., 2006; Hebert et al., 2013), characterized by progressive episodic memory loss and other executive, linguistic, and behavioral symptoms (McKhann et al., 2011). For example, AD is more likely to occur if one leads a lonely life (Wilson et al., 2007; Cacioppo et al., 2014) or suffers from a vascular disease (Dickstein et al., 2010). Conversely, AD may be delayed or even prevented through sustained stimulating activities. For instance, the onset of dementia is considerably delayed in individuals who have higher educational and occupational achievements (Stern, 2012) or who develop musical expertise (Hanna-Pladdy and MacKay, 2011).

The latter findings have been taken as evidence for the phenomenon of cognitive reserve (CR), the brain's capacity for functional compensation or resilience following damage or throughout healthy aging (Stern, 2012; Cabeza and Dennis, 2013). In the former sense, CR refers to the relationship between the degree of pathology or brain damage and the intensity or earliness of its clinical manifestations (Stern, 2009). In particular, the deleterious effects of brain damage would be delayed or reduced by experience-induced changes in specific neurocognitive networks (Stern, 2009). This would be favored by activities which engage circuits sensitive to age-related attrition and age-related functional activation increases (Cabeza and Dennis, 2013).

The view has recently emerged that CR may be enhanced by a specific type of linguistic experience: life-long bilingualism (Kavé et al., 2008; Bak et al., 2014). In line with evidence for a bilingual advantage in executive functioning (see Section Bilingualism: Key Notions and Findings), some studies found that symptoms of dementia (Bialystok et al., 2007, 2014; Chertkow et al., 2010; Craik et al., 2010; Alladi et al., 2013; Woumans et al., 2015) and mild cognitive impairment (MCI; Ossher et al., 2013; Bialystok et al., 2014) have a later onset in bilingual/plurilingual than in monolingual patients. Moreover, Schweizer et al. (2012) reported comparable cognitive performance between bilingual and monolingual AD patients despite greater brain atrophy in the former. Bilingualism has also been claimed to favor CR throughout healthy aging (Bak et al., 2014), especially if high proficiency levels are attained (Gollan et al., 2011). Additional studies found that life-long bilingualism is positively associated with white matter integrity (Gold et al., 2013; Olsen et al., 2015) and gray matter density (Abutalebi et al., 2014, 2015) in several brain areas, crucially including the frontal lobes.

However, the claim that bilingualism fosters CR is not uncontroversial. First, several studies have failed to replicate the above findings (Crane et al., 2010; Sanders et al., 2012; Clare et al., 2014; Zahodne et al., 2014; Kowoll et al., 2015; Lawton et al., 2015). Second, non-trivial methodological caveats can be identified in the (seemingly) confirmatory reports. Finally, the evidence for volumetric brain differences between bilinguals and monolinguals does not directly imply increased CR. Moreover, it remains unclear which cognitive functions would be preserved by bilingualism and when such protective effects would become manifest.

Consequently, at present it is not clear whether bilingualism delays the onset of dementia. The main results vary from study to study, with some of them agreeing on at least 4 years of delay in AD and MCI symptoms, and others yielding no difference between monolinguals and bilinguals. It may well be the case that bilingualism contributes to CR in later-life. Yet, for this view to be fully embraced we must explicitly address contradictory findings, identify possible lurking variables, and outline methodological alternatives thereof. The present paper pursues those three goals. First, we review the available evidence, highlighting its inconsistencies and methodological differences. Second, we characterize possible confounding factors that may have influenced the results. Our focus is on the criteria to establish bilingualism, differences in sample design, the instruments used to examine cognitive skills and arrive at a diagnosis, and the role of variables known to modulate life-long cognition. Finally, we propose that these limitations may be partly circumvented by expanding the methodological toolkit used so far and by adopting experimental rather than retrospective approaches. Such considerations may illuminate the relationship between bilingualism and CR, as well as the development of compensation mechanisms throughout aging in general.

## Bilingualism: Key notions and findings

The term “bilingualism” has received various definitions in the specialized literature. Whereas some authors have used it restrictively to mean “native-like mastery of two languages” (e.g., Bloomfield, 1935), others employ it more broadly as the alternate use of two languages, irrespective of proficiency (e.g., Weinreich, 1953; Mackey, 1968). In line with the latter view, here we will subscribe to Grosjean's (1994) definition of a bilingual as any person who uses two languages or dialects in daily life. Within this broad population, bilinguals can be classified in terms of age of second language (L2) acquisition (early vs. late bilinguals), simultaneity of L2 acquisition (simultaneous vs. sequential bilinguals), L2 proficiency (from incipient to low-, mid-, and high-proficiency bilinguals), and frequency of L2 use (active vs. latent bilinguals), among other variables. Note that the notion of bilingualism is sometimes taken as synonymous with plurilingualism (i.e., sustained use of more than two languages). However, both need to be differentiated. Indeed, cognitive performance in several domains (Kavé et al., 2008), including inhibitory control (Marian et al., 2013), is modulated by the acquisition of languages beyond the L2. Thus, the term “bilingual” should be reserved to individuals who possess daily functional skills in only two languages.

Neuroanatomically, a native language (L1) and an L2 are subserved by independent neural networks, although these may be located in shared gross regions, as indicated by aphasiological (Paradis, 2004, 2009), electrostimulation (Ojemann and Whitaker, 1978; Rapport et al., 1983), and neuroimaging (Chee et al., 2003; Klein et al., 2006) studies. During verbal production, language selection and inhibition of lexical competitors implicate both cortical (left prefrontal cortex) and subcortical (anterior cingulate cortex, left caudate nucleus, bilateral supramarginal gyri) structures (Abutalebi and Green, 2008). Switching between both languages depends on a broad bilateral frontotemporal network, as shown by a recent meta-analysis (Luk et al., 2012). However, a bilingual's neurocognitive profile is sensitive to L2-related variables, such as age of acquisition, exposure, and proficiency. For example, the functional and anatomical correlates of the L1 and the L2 tend to be more similar in early than in late bilinguals, especially if L2 exposure is constant (Ardal et al., 1990; Neville et al., 1992, 1997; Perani et al., 1996; Kim et al., 1997; Weber-Fox and Neville, 1997; Ullman, 2001; Paradis, 2009). Similarly, the neurofunctional mechanisms engaged by each language are typically more convergent in high-than in low-proficiency bilinguals (e.g., Perani et al., 1998; Videsott et al., 2010), as confirmed by a meta-analysis of 14 neuroimaging studies (Sebastian et al., 2011).

Another line of research has assessed how bilingualism impacts cognitive functions across multiple domains. Bilinguals have been observed to present advantages in certain aspects of executive functioning, such as inhibitory control and working memory (for a review, see Bialystok et al., 2009). Such effects have been reported in children (Carlson and Meltzoff, 2008; Adi-Japha et al., 2010; Bialystok, 2011), young adults (Rodriguez−Fornells et al., 2006; Costa et al., 2008; Prior and MacWhinney, 2010), and older adults (Bialystok et al., 2004; Salvatierra and Rosselli, 2010). In this vein, a meta-analysis of 63 studies reported that bilingualism was associated with increased attentional control and working memory skills, among other domains (Adesope et al., 2010). By way of explanation, it has been proposed that the more stringent language control demands faced by bilinguals during everyday communication enhance domain-general executive functioning. This view is further supported by evidence that professional simultaneous interpreters outperform non-interpreter bilinguals certain executive measures, such as working memory tasks (Bajo et al., 2000; Christoffels et al., 2006; Yudes et al., 2011).

However, claims for a bilingual advantage have recently come under fire. First, some non-executive domains, such as single-language receptive vocabulary and fluency, consistently reveal bilingual disadvantages (Bialystok et al., 2009). Moreover, several recent comparisons of executive performance between bilinguals and monolinguals have mostly yielded null results (for a review, see Duñabeitia and Carreiras, 2015). It has even been shown that cognitive advantages of bilinguals relative to monolinguals may be eliminated depending on the data trimming procedure (Zhou and Krott, 2015). In addition, the evidence of enhancements induced by interpreting expertise is not entirely robust (for a review, see García, 2014a). Finally, studies on possible neuroanatomical changes associated with the bilingual experience have yielded ambiguous results (García-Pentón et al., 2015). Thus, although claims for distinctive neurocognitive effects of bilingualism have attracted great scholarly and media attention in recent years, accumulating data reveals a hazy and inconsistent picture. As we argue below, the same seems to be true of studies on bilingualism and CR.

## Cognitive reserve in bilinguals with dementia: An inconsistent body of data

The main claims for a protective effect of bilingualism stem from retrospective analyses of clinical records. Although recent studies have explored the issue by considering motor diseases—e.g., Parkinson's disease (Hindle et al., 2015)—, the bulk of the evidence comes from comparisons between monolingual and bilingual AD patients. Bialystok et al. (2007) first observed that dementia appeared roughly 4 years later in 184 bilingual immigrants who spoke English and any other language. A similar finding was reported by Craik et al. (2010) in a sample including 211 immigrants and non-immigrants. A delay of approximately 4.5 years was also observed in 134 elderly AD patients including non-immigrant bilinguals (Woumans et al., 2015). This pattern was replicated in 648 subjects with mixed types of dementia (Alladi et al., 2013) and 149 patients with both MCI and AD (Bialystok et al., 2014). Also, a study controlling for childhood intelligence in 853 healthy individuals found that elderly bilinguals had better cognitive performance than predicted from their baseline abilities (Bak et al., 2014).

However, other studies have only partially supported the hypothesis, as they found confirmatory evidence only for specific bilingual subgroups. Chertkow et al. (2010) compared the age of symptom onset and diagnosis in 632 subjects who were monolingual, bilingual, and plurilingual AD patients. They reported a delay of 3 years for immigrant bilinguals and plurilinguals, but no significant benefit in non-immigrant bilinguals. Moreover, a protective effect was observed for non-immigrants whose L1 was French, but not for those whose L1 was English. Ossher et al. (2013) examined 111 patients with MCI and observed a symptom-onset delay only for amnestic bilinguals. Also, a study on executive functions (inhibition, attention, and working memory) with healthy elderly participants found that bilingual advantages were restricted to highly proficient individuals (Gollan et al., 2011). In addition, Kousaie and Phillips (2012) used a Stroop task in a non-immigrant sample of 118 young and older monolinguals and bilinguals and showed that only bilingual young adults had a general speed advantage relative to their monolingual counterparts, but this was not associated with smaller Stroop interference.

Crucially, several longitudinal studies on AD found no evidence for increased CR in bilinguals. In the report by Crane et al. (2010), 2520 second-generation Japanese-Americans (non-demented at baseline) were assessed for dementia on three occasions over 6 years. Midlife use of spoken and written Japanese was not related to lower cognitive decline rates in later life. Another study (Sanders et al., 2012) compared the incidence of dementia in 1779 elderly native and non-native English users. The latter group gave no evidence of increased CR, and actually exhibited a small (yet not significant) increase in risk for dementia. Notably, non-native speakers with at least 16 years of education had a four-fold increased risk for dementia compared to less educated participants. Similarly, Zahodne et al. (2014) tested 1067 AD participants at 18–24 month intervals for up to 23 years. Almost 300 subjects developed dementia in the course of the study. Bilingualism was associated with better memory and executive function at baseline. However, it was not related to rates of cognitive decline or dementia conversion. Finally, Kowoll et al. (2015) did not observe significant neuropsychological differences between monolingual and bilingual MCI/AD patients in a sample of 86 participants. However, the authors concluded that the dominant language may be compromised first in bilingual MCI patients, while severe deficits of the non-dominant language would appear later, when AD becomes manifest.

Null results have also been reported in cohort studies. Clare et al. (2014) conducted a cross-sectional investigation with 86 early AD patients. At the time of diagnosis, bilinguals were on average 3 years older than monolinguals, but they also exhibited significantly greater cognitive deficits. Moreover, despite relatively better performance on inhibition and response conflict tasks, bilinguals possessed no significant advantages on executive function. Additional evidence comes from the cohort study by Lawton et al. (2015). They assessed 1789 Hispanic Americans above age 60 (half of whom were immigrants) every 12–15 months for 10 years. Fifty-five participants were diagnosed with AD and 26 with vascular dementia. Crucially, mean age of diagnosis was not significantly different among bilingual and monolingual (U.S.-born or immigrant) patients. For further details on these studies, see Table 1.

**Table 1:** **Summary of retrospective studies on the relationship between bilingualism and cognitive reserve in demented populations**.

**Study**	**Sample**	**Asst of bilingualism and lg prof**	**Control of variables affecting CR**	**Asst of dementia or cognition**	**Results**
Bialystok et al., 2007	**91 MLs with AD** (48 f) Age of onset: 71.4 Age at first app: 75.4 Years of education: 12.4 MMSE at first app: 21.3 Occupation status^*^: 3.3 **93 BLs with AD** (55 f) (all I: E and any other lg) Age of onset: 75.5 Age at first app: 78.6 Years of education: 10.8 MMSE at first app: 20.1 Occupation status^*^: 3.0	Subjective lg interview; E fluency; place of birth; date of birth; year of IMG	Years of education and occupation status. Some patients had CeVD, depression psychosis, meningioma, or sleep apnea	MMSE; CT; SPECT	**More CR in BLs** BLs were at least 4 years older at disease onset
Chertkow et al., 2010	**379 MLs with AD** (240 f) (23I: 66 spoke F, 290 spoke E) Age at diagnosis: 76.7 Years of education: 10.9 MMSE score: 23.1 **253 PLs with AD** (131 f) (135 I, 19 NI with E as L1) Age at diagnosis: 77.6 Years of education: 10.7 MMSE score: 22.9	Patient and caregivers interviews. IMG/native status assumed. AoA and age of IMG not controlled	Same as Bialystok et al. (2007)	MMSE	**More CR in some BLs** Three-year delay for I BLs and PLs. No benefit in NI BLs. Benefit for NI with F as L1, but not for E as L1
Craik et al., 2010	**109 MLs with AD** (60 f) Age at onset: 72.6 Age at first app: 76.5 Duration: 3.8 Years of education: 12.6 MMSE at first app: 21.5 Occupation status^*^: 2.8 **102 BLs with AD** (60 f) Age at onset: 77.7 Age at first app: 80.8 Duration: 3.1 Years of education: 10.6 MMSE at first app: 20.4 Occupation status^*^: 2.5	Same as Bialystok et al. (2007)	Same as Bialystok et al. (2007)	MMSE	**More RC in BLs** BLs had been diagnosed 4.3 years later and reported the onset of symptoms 5.1 years later
Crane et al., 2010	**2520 2nd generation J-A BLs cf**. (all men) **465 Neither spoke nor read J** Age at HAAS Exam. 4: 76.1 Education: 11.1 Standard CASI score at HAAS Exam 4: 89/100 **1495 spoke but did not read J** Age at HAAS Exam 4: 76.6 Education: 10.8 Standard CASI score at HAAS Exam 4: 87.4/100 **560 both spoke and read J** Age at HAAS Exam 4: 77.5 Education: 11.5 Standard CASI score at HAAS Exam 4: 87	Two questions on oral and written skills in J	Education, AI, category, blood sample analyzed for APOE, head circumferences, smoking status (never, past, current)	CASI: at HAAS baseline (ages 71–93) and three more waves: HAAS Exams (ages 74–95, 77–98, 79–100); DSM, IRT	**Similar CR** No evidence to support the CR hypothesis Rates of cognitive decline not related to use of spoken or written J
Gollan et al., 2011	**22 high educated BLs with AD** Age of diagnosis 75.1 Age of onset: 72.1 Education: 14.6 MMSE: 23.4 BNT-based Bilingual Index+: 64 Self-rated Bilingual: 74 % daily use of E: 75.6 AoA of E: 2.9 **22 low-educated BLs with AD** Age of diagnosis 77.1 Age of onset: 75.0 Education: 5.6 MMSE: 24.2 BNT-based Bilingual Index:.42 Self-rated Bilingual Index:.52 % daily use of E: 21.1 AoA of E: 19.9	Objective measure: calculated bilingual index scores through BNT, compared that score to an index of BLs' self-rated spoken prof in each lg	Education, degree of bilingualism	MMSE, DRS, BNT	**More CR in some BLs** Higher degrees of bilingualism associated with later age-of-diagnosis, but only in P with low education level (most were S-dominant). Only objective measures, predicted age-of-diagnosis
Sanders et al., 2012	**1389 NES** (845 f) Age: 78.3 Dementia incidence rate: 2.87 Race: (*n* white) 928 Education: 13.3 Reading grade level: 12 Immigrated to united states: 155 Married: 531 BIMC: 2 FCSRT: 29.9 15-item GDS: 2 **390 n.NES** (236 f) Age: 79.4 Dementia incidence rate: 3.54 Race: (*n* white): 317 Education:12.5 Reading grade level: 12 Immigrated to united states: 243 Married: 6 BIMC: 2 FCSRT: 30.1 15-item GDS: 2	One question at baseline about E as L1 n-NES further asked to define their L1, the age at which they learned E, and frequency of E usage.	Self-reported education, self-reported medical hx (diabetes, HTN, stroke) IMG,	DSM-IV criteria, BIMC, pre- morbid intelligence, attention, episodic memory, EF, visuospatial ability and lg, 15-item GDS	**Similar CR** No protective effect against AD related to n.NES status (maybe in an education-dependent manner)
Schweizer et al., 2012	**20 MLs with AD** (14 f) Age at CT scan: 77.2 Age at diagnosis: 77.3 Education: 13.6 Occupation status^*^: 3.2 MMSE:23.2 **20 BLs with AD** (14 f) Age CT scan: 78.9 Age at diagnosis:78.9 Education: 11.6 Occupational status^*^: 2.1 MMSE: 22.1	Bilingualism was confirmed by a spouse or caregiver in most patients	Education (groups were similar), occupation (groups differed)	MMSE CDR, Katz ADL index, BNA, and the CDT	**More RC in BLs** BLs showed greater brain atrophy in the radial width of the temporal horn and the temporal horn ratio
Kousaie and Phillips, 2012	**38 young healthy MLs** Age: 22.5 Education: 15.1 MoCA: 28.6 **35 young healthy MLs** Age: 23.7 Education: 15.5 MoCA 27.8 **25 Older healthy MLs** Age: 68.9 Education: 13.9 MoCA: 26.8 **20 Older healthy BLs** Age: 71.9 Education: 15.9 MoCA: 26.6	Animacy judgment task to assess relative L1/ L2 lg prof. Self-asst of listening, reading, and speaking in each lg on a scale of 15-	Education	MoCA, Stroop test	**No relation between bilingualism and CR** Young adult BLs showed a general speed advantage relative to MLs (not associated with smaller Stroop). Older adults showed no effect of bilingualism
Alladi et al., 2013	**257 MLs with AD** (126 f) Age at onset: 61.1 Age at pres: 63.4 Years of education: 5.9 Occupation: 107 Literacy: 177 Urban: 135 Duration: 2.1 MMSE. 16.7 **391 BLs with AD** (98 f) Age at pres: 68.1 Age at onset: 65.3 Years of education: 12.9 Occupation: 257 Literacy: 373 Urban: 292 Duration: 2.3 MMSE. 18.9	Subjective interview to a fam member	Sex, literacy, years of education, occupation, urban vs. rural dwelling, age at pres, age at onset, duration of the illness, MMSE score, ACE-R, CDR, dementia subtype (FTD, VaD, DLB, mixed), fam hx of dementia, VRF, HTN, diabetes, smoking, alcoholism, CAD, and stroke	MMSE ACE-R CDR (mild, moderate, severe)	**More CR in BLs** BLs were older, more educated, had higher skill levels in their occupation, etc. BLs presented dementia symptoms 4.5 years later than MLs
Ossher et al., 2013	**49 MLs with single-domain aMCI** (55% f) Age: 74.9 Duration of symptoms: 2.4 Education: 14.7 MMSE: 27.7 **19 BLs with single- domain aMCI** (32% f) Age: 79.4 Duration of symptoms: 2.8 Education: 14.5 MMSE: 27.6 **22 MLs with multiple-domain aMCI** (45% f) Age: 75.2 Duration of symptoms:2.7 Education: 14.9 MMSE: 27.9 **21 BLs with multiple-domain aMCI** (43% f) Age: 72.6 Duration of symptoms:2.0 Education: 15.0 MMSE: 27.7	QNR: L1 acquired, other lgs spoken, frequency, etc. All patients proficient in E, but BLs spoke a variety of other lgs and were not drawn from any single specific sociocultural group.	Social background, Education	MMSE Two Tests from the D-KEFS, vocabulary, HVTL (total and delayed recall), digit span, BNT, ROCF, TMT, Stroop, VF	**More RC in BLs (only in one aMCI type)** Only those with single-domain aMCI had a later age of diagnosis for BLs (79.4 years) than MLs (74.9 years**)**
Zahodne et al., 2014	**637 initially non-demented MLs** (72 f) Baseline age: 75.66 Education: 5.05 Age of IMG: 48.23 **430 initially non-demented BLs** (64 f) Baseline age: 74.78 Education: 8.30 Age of IMG: 34.22	Self-report and WRAT 3	Education, IMG	DSM III, SRT, 15-item BNT, WAIS revised, CTT	**Similar CR** No protective effect of bilingualism
Clare et al., 2014	**49 MLs with AD** (22 f) Age at testing: 78.82 Education: 12.31 CR: 97.79 Subjective health status: 72.17 **37 BLs with AD** (21 f) Age at testing: 80.76 Education: 11.84 CR: 100.45 Subjective health status: 73.5	Lg QNR. NART (revised) BNT, Spot-the-Word Test, BPVS, the Prawf Geirfa Cymraeg i Oedolion. Some BLs spoke W from birth and others around age 4–5. The age at which they began to speak E varied	Education occupation, socio-economic status, CR, subjective health status, Anxiety, depression, AChEI medication	MMSE, background measures, lg prof, EF	**Similar CR** BLs with AD came to the attention service later. No significant differences btw MLs and BLs in EF tests, but BLs showed relative strengths on inhibition and response conflict
Bialystok et al., 2014	**38 MLs with MCI (19 f, 12 I)** Onset age: 62.2 Clinic age: 66.5 Years of education: 15.5 BNA: 95.4 MMSE: 29.0 **36 BLs with MCI (20 f, 25 I)** Onset age: 66.9 Clinic age: 70.0 Years of education: 14.3 BNA: 90.6 MMSE: 28.4 **35 MLs with AD (19 f, 8 I)** Onset age: 70.9 Clinic age: 74.2 Years of education: 12.5 BNA: 72.7 MMSE: 23.4 **40 BLs with AD (22 f, 27 I)** Onset age: 78.2 Clinic age: 81.4 Years of education: 12.2 BNA: 63.8 MMSE: 22.3	LSBQ. All patients were proficient in E, but BLs spoke a variety of other lgs and did not represent any single specific sociocultural group. Some P spoke more than two lgs, but were included in the BLs group	Diet Smoking P.A Social activity IMG hx Education	MMSE BNA Three EF Tasks from the D-KEFS	**More RC in BLs** BLs reported later onset ages than MLs for both the MCI group (by 4.7 years) and the AD group (by 7.3 years)
Bak et al., 2014	**853 healthy BLs (410 f, learned L2 during study)** Age: 72.49	QNR administered to P: learning of any L2, AoA, number lgs, frequency of use in: conversation/reading/media Only few P acquired their L2 before age 11	—	Intelligence, memory, WAIS III, Moray house test, NART, VF.	**More RC in BLs** A protective effect of bilingualism against age-related cognitive decline independently of CI. Knowing 3 or more lgs produced stronger effects than knowing 2
Woumans et al., 2015	**69 MLs with AD (48 f)** Age: 76.4 Mann Age: 73.0 Diagnosis age: 73.8 Initial MMSE: 24.2 Education: 13.5 **65 BLs with AD (45 f)** Age: 77.9 Mann age: 74.3 Diagnosis age: 75.5 Initial MMSE: 23.8 Education: 14.7	Patient and caregiver subjective interviews; AoA; prof, usage	Education, occupation and socioeconomic status	Heteroanamnesis, physical exam, MMSE screening blood tests, SPECT, PET, CT, and/or MRI	**More RC in BLs** Age of AD manifestation was 71.5 in MLs and 76.1 in BLs
Lawton et al., 2015	**54 MLs with AD (65% f)** Age of dementia diagnosis: 81.1 Years of education: 4.99 3MSE score: 78.87 type 2 diabetes: 46% S speaking: 76% E speaking: 24% **27 BLs with AD (63% f)** Age of dementia diagnosis: 79.3 Years of education: 7.70 3MSE score: 79.56 Type-2 diabetes: 53%	Two questions from the “ARSMA-II” (lg/s spoken). Answers were coded on a 0–3 point Likert scale	IMG, education, diabetes,	3MSE, S and E Verbal Learning Test, SENAS, IQCODE	**Similar CR** BLs much better educated, but U.S.-born BLs and MLs did not differ. Mean age of dementia diagnosis was similar in both groups
Kowoll et al., 2015	**11 BL controls (7 f)** Age: 68.2 MMSE: 29.0 Education: 14.2 **6 ML controls (2 f)** Age: 70.2 MMSE: 29.6 Education: 12.2 **8 BLs with MCI (3 f)** Age: 71.3 MMSE: 26.8 Education: 13.4 **14 MLs with MCI (5 f)** Age: 77.5 MMSE: 26.4 Education: 12.2 **22 BLs with AD (11 f)** Age: 77.2 MMSE: 22.0 Education: 12.5 **25 MLs with AD (9 f)** Age: 80.3 MMSE: 18.9 Education: 9.5	Self-rating scale of 1–7; lg hx QNR(AoA, prof level, etc.); BTN, verbal fluency task from the CERAD Comparison between objective and subjective measures similar to Gollan et al. (2011)	Education, IMG, lg dominance	CERAD-NP, MMSE, TMT, clock drawing test, WMS-R and WMS IV(), GDS	**Similar CR** Dominant lge may be firstly compromised in bilingual MCI patients. Deficits of the non-dominant language later in the course of AD

*The table features all available data for each variable in each study. CR, cognitive reserve; AD, Alzheimer's disease; MLs, monolinguals; BLs, bilinguals; PLs, plurilinguals; f, female; n, number; P, participants; prof, proficiency; man, manifestation; QNR, questionnaire; app, appointment; IMG, immigration; I, immigrant; NI, non-immigrants; P.A, physical activity; CT, computed tomography; SPECT, single-photon emission computed tomography; L1, first language, L2, second language; lg, language; Asst, assessment; CI, childhood intelligence; pres, presentation; NES, native English speaker; n.NES, non-native English speakers; F, French; S, Spanish; E, English; J, Japanese; A, American; W, Welsh; Exam, examination; AI, annual income; EF, executive function; MMSE, Mini-Mental-Status Examination; 3MSE, modified Mini-Mental-Status Examination; IQCODE, Informant Questionnaire on Cognitive Decline in the Elderly; LSBQ, Language and Social Background Questionnaire; WRAT 3, Wide Range Achievement Test; D-KEFS, Delis-Kaplan Executive Function System; SRT, Selective Reminding Test; BNT, Boston Naming Test; WAIS, Wechsler Adult Intelligence Scale; ROCF, Rey-Osterreich Complex Figure Copy; TMT, Trail-Making Test; CTT, Color Trial Test; MoCA, Montreal Cognitive Assessment; NART, National Reading Adult Test; APOE e 4 alleles, Apolipoprotein E; CASI, Cognitive Abilities Screening Instrument; HAAS, Honolulu-Asia Aging Study; IRT, Item Response Theory; BIMC, The Blessed Information-Memory-Concentration test; FCSRT. Free and Cued Selective Reminding Test; CDR, Clinical Dementia Rating; ACE-R, Addenbrooke's Cognitive Examination-Revised; DSM, Diagnostic and Statistical Manual of Mental Disorders; CDT, the Clock Drawing Test; Katz ADL, Activities of Daily Living; BNA, Behavioral Neurology Assessment; GDS, Geriatric Depression Scale; BPVS, British Picture Vocabulary Scale; ARSMA, Acculturation Rating Scale for Mexican Americans; SENAS, Spanish-English Neuropsychological Assessment Scale; CAD, Coronary Art Disease; HTN, Hyperthension; fam, family; hx, history; DLB, dementia with Lewy bodies; FTD, fronto-temporal dementia; VaD, vascular dementia; CeVD, cerebrovascular disease; aMCI, amnestic mild cognitive impairment*.

* *Occupation was determined through the system developed by Human Resources and Skills Development, Canada (2001). Occupations are classified on a five-point scale, with higher numbers associated with higher status*.

In sum, positive and null results have been evenly documented in the literature. This suggests that bilingualism may contribute to CR, but only under certain unknown conditions. To a large extent, such discrepancies may reflect methodological differences between, and shortcomings within, the available studies. Such factors are discussed below in an attempt to foster more robust approaches to the issue.

## Methodological caveats within and across studies

Non-experimental research faces important caveats when it targets a population as diverse as bilinguals, especially those affected with heterogeneous conditions such as AD. The impossibility to control critical factors and collect relevant subject data leads to widespread intra- and inter-group variability. In this sense, the above studies are characterized by major differences and/or limitations concerning four factors: (i) the conception of bilingualism and the assessment of language proficiency, (ii) sample design, (iii) the instruments used to assess cognitive skills and diagnose the underlying clinical entity, and (iv) the examination of other variables known to affect CR.

### Shortcomings in the conception and assessment of bilingualism

The literature presents several caveats regarding the conception of bilingualism and the assessment of language proficiency. Most of the retrospective studies in Section Cognitive reserve in Bilinguals with Dementia: An Inconsistent Body of Data established bilingualism and proficiency via subjective interviews with the patients (Bialystok et al., 2007; Chertkow et al., 2010; Craik et al., 2010; Crane et al., 2010; Sanders et al., 2012; Woumans et al., 2015) or their caregivers (Chertkow et al., 2010; Craik et al., 2010; Schweizer et al., 2012; Alladi et al., 2013; Woumans et al., 2015). However, subjective estimations of proficiency can be unreliable and biased by self-perception (Hulstijn, 2012). In some studies (e.g., Chertkow et al., 2010), data concerning L2 acquisition and immigration status were unavailable and thus impressionistically estimated.

Likewise, age of acquisition varied greatly among and even within studies. In the case of Woumans et al. (2015), for instance, some participants had acquired their L2 since birth, others around puberty, and still others during adulthood. This constitutes another potential confound, given that bilinguals rely on different cognitive mechanisms depending on the manner and age of L2 appropriation—viz., incidental acquisition vs. metalinguistic learning (Paradis, 2009)—, as well as the sociocultural circumstances framing bilingual development—e.g., circumstantial vs. elective bilingualism in immigrants and L2 learners, respectively (Valdés and Figueroa, 1994).

Moreover, most reports confounded bilingualism with plurilingualism. For instance, in the study by Alladi et al. (2013), 26.2% of the participants spoke two languages, whereas more than 34% spoke three or more languages. This is a non-trivial consideration, since the neurocognitive resources taxed during bilingual processing are sensitive to the presence of additional languages (Marian et al., 2013). Indeed, a study comparing bilinguals, trilinguals, and plurilinguals (Kavé et al., 2008) showed that the number of non-native languages spoken influenced cognitive performance beyond the effect of demographic variables.

Also, most of the studies included bilinguals possessing varied language pairs, only some of which were typologically similar (e.g., Kowoll et al., 2015; Woumans et al., 2015). However, access and control mechanisms in bilingual processing differ depending on the typological distance between languages (Tao et al., 2011; García, 2014b). Moreover, assessments of monolingualism are absent in most studies. This is a critical aspect for the literature on bilingualism and CR, as there may not be such a thing as a “pure monolingual” (De Bot and Jaensch, 2015).

All in all, the literature on bilingualism and CR proves inconsistent and sometimes flawed in its characterization of the former variable. To a large extent, this is due to the reliance on retrospective, non-experimental approaches, which precludes the construction of carefully controlled samples. It is crucial to for the field to develop more robust sampling procedures, especially in the exploration of possible neurological correlations. Indeed, L2 proficiency positively correlates with gray matter volume in control-relevant areas (Stein et al., 2012) and with the age of AD diagnosis and symptom onset (Gollan et al., 2011).

### Variability in sample design

Proper randomization is not easily achieved in retrospective designs, and sample sizes vary greatly across studies. In the present review, three studies included less than 50 participants (Gollan et al., 2011; Schweizer et al., 2012; Kowoll et al., 2015), two less than 100 (Clare et al., 2014; Lawton et al., 2015); six less than 250 (Bialystok et al., 2007, 2014; Craik et al., 2010; Kousaie and Phillips, 2012; Ossher et al., 2013; Woumans et al., 2015), three between 500 and 1000 (Chertkow et al., 2010; Alladi et al., 2013; Bak et al., 2014), and another three between 1000 and 2500 (Crane et al., 2010; Sanders et al., 2012; Zahodne et al., 2014). Since effect sizes are partially linked to sample sizes, especially when effects are robust (Little, 2013), these differences across studies may partly account for their discrepant findings.

In retrospective studies, as in experimental designs, demographic (e.g., age, sex, education) and clinical (e.g., other pathologies) variables must be matched between compared groups to adjust for potential confounds and increase precision in the analysis. In this sense, a further caveat of the studies is that they compare samples of different sizes. In some cases, there were 50% more monolinguals than bilinguals (e.g., Chertkow et al., 2010; Zahodne et al., 2014; Lawton et al., 2015), and in the study by Sanders et al. (2012) one of the groups tripled the other in size. Samples with such different sizes may lead to biased results, as the larger one is likely to feature greater variance and thus be more representative of its respective population. This is especially true when the smaller group is the one composed by bilinguals, as random selections of such individuals may not realistically represent the wide spectrum of ages of acquisition, levels of competence, and degrees of exposure.

This scenario is further complicated by sample heterogeneity. Some studies compared monolinguals and bilinguals, others examined different types of bilinguals, and still others considered different types of dementia, sometimes without control group (see Table 1 for details). In particular, dementias are not homogeneous conditions, and they may differ in their susceptibility of early diagnosis, progression speed, degree of genetic compromise/vulnerability, and the local or global nature of affected cognitive domains. In addition, inclusion and exclusion criteria in most studies are either absent or poorly specified. Finally, these studies fail to complement measures of statistical significance (*p*-value) with calculations of effect size. This would be useful to ascertain the minimum number of participants needed to avoid a Type II, or β, error (Sullivan and Feinn, 2012). Importantly, sample size should be established before initiating any study and, as far as possible, it should not be changed during the course of the study (Kadam and Bhalerao, 2010). In this respect, only few studies have contemplated these measures (Bak et al., 2014; Bialystok et al., 2014; Clare et al., 2014; Woumans et al., 2015), and some have excluded participants who were not relevant for the analysis, changing the size of the initial sample (Chertkow et al., 2010; Crane et al., 2010; Gollan et al., 2011; Sanders et al., 2012; Zahodne et al., 2014; Lawton et al., 2015).

All in all, there is major variability in sample size calculation and stability. Also, some crucial statistical aspects have not been made explicit enough in the literature. Retrospective studies may lack power to achieve significance but these measures should at least be mentioned (Sullivan and Feinn, 2012).

### Reservations on the instruments used to assess cognitive deficits and diagnose the underlying clinical entity

Importantly, there is variability in the criteria for the diagnosis of MCI and AD. All but one of the 14 studies targeting pathological groups established diagnosis through the consensus of medically qualified clinical staff. In seven of them (Bialystok et al., 2007, 2014; Chertkow et al., 2010; Craik et al., 2010; Sanders et al., 2012; Kowoll et al., 2015; Lawton et al., 2015), AD was diagnosed following NINCDS-ADRDA criteria (McKhann et al., 1984); two followed the DSM III/IV criteria (Crane et al., 2010; Sanders et al., 2012, respectively); one relied on ICD-10 (Clare et al., 2014), and three followed other criteria (Schweizer et al., 2012; Alladi et al., 2013; Clare et al., 2014) in the absence of clinical staff consensus.

In addition to these diagnostic discrepancies, the underlying pathology was not consistent among or even within some studies. Ossher et al. (2013) focused on patients with MCI, as established via the criteria in Petersen (2004). This condition was considered in tandem with AD by Bialystok et al. (2014), who followed the diagnostic criteria proposed by Albert et al. (2011). Also, for 130 patients in the study by Chertkow et al. (2010), dementia onset was defined as the clinic visit at which a preceding MCI diagnosis changed to AD. Moreover, the study by Bialystok et al. (2007) included 52 patients diagnosed with other dementias (including possible AD). These differences also cast further doubt on the consistency of the results, given that diagnosis can vary greatly depending on the type of impairment exhibited by patients and on the criteria used to establish the underlying clinical entity (Burvill, 1993).

Further reservations concern the instruments used to assess dementia. Virtually all studies have done so via the MMSE (Folstein et al., 1975). Whereas some have incorporated additional measures—e.g., the Behavioral Neurology Assessment (Schweizer et al., 2012; Bialystok et al., 2014), the Clinical Dementia Rating (Schweizer et al., 2012; Alladi et al., 2013), the Addenbrooke's Cognitive Examination-Revised (Alladi et al., 2013)-, in most cases this was the only neuropsychological measure employed (e.g., Bialystok et al., 2007; Chertkow et al., 2010; Craik et al., 2010; Kowoll et al., 2015; Woumans et al., 2015). However, abundant research shows that the MMSE is of limited value to diagnose onset dementia (Wind et al., 1997; Kim and Caine, 2014), measure its progression within periods of less than 3 years (Clark et al., 1999), detect MCI (Tang-Wai et al., 2003), and even assess general cognitive profile (Feher et al., 1992). Actually, it is possible to have AD and still score 30/30 on this test (Shiroky et al., 2007). In addition, initial symptoms of AD could be even more difficult to detect in individuals with higher CR.

Furthermore, neither the MMSE nor the other instruments used are specifically aimed at assessing executive domains, whose evaluation may be critical to ascertain the specific impact of bilingualism on CR (Bialystok et al., 2014). Note, also, that executive dysfunction may represent one of the early manifestations of AD. Thus, the inclusion of additional, sensitive measures (especially those targeting executive performance) could prove crucial for the field to progress.

### Is it really bilingualism? Lurking variables known to affect CR

Claims for a specific relationship between bilingualism and CR must rule out the influence of confounding factors. Available studies have only partially succeeded in this regard. While most of them offer data on the patients' age, education level, and socioeconomic status (SES), they rarely contemplate other relevant factors.

CR may be enhanced by varied habits and personality traits. For instance, it may be promoted through social networking (Bennett et al., 2006) or sustained intellectual stimulation across the lifespan (rather than education; Scarmeas and Stern, 2004). Other contributing factors are overall fitness, amount of exercise, and type of sustained physical activity (e.g., Davenport et al., 2012). In particular, healthy aerobic exercise reduces hippocampal volume loss and improves memory in adulthood (Erickson et al., 2011). CR may also be enhanced by the development of emotional skills favoring adaptive behaviors and resilience in the face of stress (Staudinger et al., 1993). Such skills may figure more prominently in immigrant than in non-immigrant individuals; thus, the conclusions advanced in some studies (e.g., Bialystok et al., 2007; Chertkow et al., 2010; Craik et al., 2010; Kowoll et al., 2015) may have been misattributed or overgeneralized—i.e., they may not be applicable for any type of bilingual.

On the other hand, some poorly controlled factors can be detrimental to CR. In particular, some of them may play a distinctive role in bilingual populations, especially in immigrant groups. Importantly, one of the most studied factors known to enhance CR is SES, which has been analyzed in most studies with bilingual immigrant groups. In this regard, high SES has been associated with reduced risk of MCI/AD (Sattler et al., 2012); however, migration often involves a loss of SES and increased rates of mental illness (Bhugra and Becker, 2005).

Moreover, immigrant populations are at increased risk for congenital and acquired neurological disorders (White et al., 2005; Zahuranec et al., 2006), alcohol abuse due to the stress of acculturation (Caetano et al., 2008; Szaflarski et al., 2011), eating disorders (Geller and Thomas, 1999; Bulik et al., 2006), poor sleep quality (Voss and Tuin, 2008), and the acquisition of bad health habits, like smoking (Bethel and Schenker, 2005). At the same time, sustained use of more than one language has been linked to greater chances of alcohol use in adolescence (Epstein et al., 1996) and to fewer acknowledgments of the dangers associated with smoking (Unger et al., 2000). Differences between bilinguals and monolinguals in these variables may further underlie discrepancies in the literature. Hippocampal neurogenesis decreases in excessive drinkers (Stevenson et al., 2009) and possibly in teetotalers (den Heijer et al., 2004). Instead, moderate consumption may favor acetylcholine release in the hippocampus (Henn et al., 1998) and thus reduce risk of AD. Also, cigarette smoking accelerates cortical thinning, a robust biomarker of cognitive decline (Karama et al., 2015). Memory impairments are also associated to other bad health habits, like poor sleep or eating disorders (Green and Rogers, 1998; Walker and Stickgold, 2006), among several others.

However, these factors have been barely controlled in the reviewed literature. Some of them have been considered with varying degrees of robustness. The most objective data have been offered by Schweizer et al. (2012), who applied the Katz Activities of Daily Living index. Bialystok et al. (2014) used a self-assessment questionnaire to glean data on the patients' diet, alcohol use, smoking habits, and physical and social activity; this approach is less reliable due to the biases inherent in self-reports of socially sanctioned practices. For their part, Alladi et al. (2013) interviewed patients' relatives to gather information on vascular risk factors, diabetes, smoking, and alcoholism (although no further methodological specifications are offered on how data were collected). Results in this study may have also been biased by the use of a heterogeneous sample of highly educated older bilinguals. Indeed, disease development may be slower and more sensitive to factors other than bilingualism in older than in younger participants. More critically, none of the studies seems to have employed instruments specifically designed to assess such variables (see Section Improving the Toolkit for Subject Sampling and Assessment).

In sum, the paucity of information regarding these factors undermines claims for and against a positive relationship between bilingualism and CR. As we propose below, future studies should more carefully consider other variables which may promote positive (e.g., synaptic strengthening) or negative (e.g., atrophy, synaptic weakening) plastic changes that may be related to MCI and AD and which may have critical effects in the bilingual population.

## A plea for methodological improvements and experimental approaches

The evidence on the relationship between bilingualism and CR is inconsistent and characterized by methodological limitations. These shortcomings, however, do not necessarily imply that CR is unaffected by the bilingual experience. We propose that the issue may be more properly addressed by refining the control of relevant subject variables and by incorporating experimental tasks.

### Improving the toolkit for subject sampling and assessment

Many of the limitations can be circumvented by adopting better instruments to assess and classify participants, with a view to maximizing homogeneity within and comparability between samples. First, the assessment of bilingualism and bilingual proficiency should be carefully considered. Questionnaires should be comprehensive enough to assess critical information, such as biographical information from participants and relatives, use of L2, proficiency, language dominance, L2 acquisition, attitude and language preference, and social status (Codó, 2008). Moreover, to maximize comparability across studies, the use of standardized questionnaires is highly advisable. A good candidate, in this sense, is the Language History Questionnaire 2.0 (Li et al., 2014). Also, to better control for L2 proficiency, it would be useful to include objective measures, such as standardized language tests or examinations in specific languages (e.g., DIALANG, Diplômes d'Études en Langue Française, Zertifikar Deutsch, Certificado de Español Lengua y Uso), vocabulary tests like LLEX (Meara, 1994), Cloze tests (Hulstijn, 2010), or even the Bilingual Aphasia Test (Paradis and Lecours, 1979; Paradis, 2011).

Second, the confounds enumerated in Section Is it Really Bilingualism? Lurking Variables Known to Affect CR could be more effectively controlled using instruments which assess them directly. For instance, the Composite International Diagnostic Interview (Robins et al., 1989) assesses habits of alcohol, tobacco, and drug use. It also considers the quality, severity, and course of substance dependence, while offering valuable information about possible impairment and comorbid mental disorders. Similarly, the Sleep Disorders Inventory (Zammit et al., 1999) has proved helpful in AD research. This instrument evaluates a wide range of sleep behaviors as well as the frequency, severity, and caregiver burden of sleeping disturbances (Tractenberg et al., 2006). Relevant data may also be gleaned through the Eating Disorders Examination-questionnaire (Fairburn and Beglin, 1994), a self-report instrument assessing restraint, weight concern, and shape concern. As these tools can help disentangle the role of lurking variables in the observed effects, future studies should include at least abridged versions of them. Furthermore, as proposed by Hogervorst et al. (2008), all patients with cognitive impairment should be assessed for hypothyroidism, as this condition correlates with lower MMSE performance at baseline, independent of FT4, age, sex, education, mood, and cardiovascular factors. In addition, given that immigrants are at increased risk for several disorders (Geller and Thomas, 1999; White et al., 2005; Bulik et al., 2006; Zahuranec et al., 2006; Caetano et al., 2008; Voss and Tuin, 2008; Szaflarski et al., 2011), non-immigrant populations should be prioritized in an attempt to establish clearer associations between bilingualism and CR (Fuller-Thomson and Kuh, 2014).

Third, more sensitive indicators of general cognitive screening should be incorporated to complement the MMSE. Robust assessments can be obtained with the traditional Mattis dementia rating scale (Mattis, 1998), the Alzheimer's Disease Assessment Scale (Mohs et al., 1983; Mohs and Cohen, 1988; Mohs, 1994), and the Montreal Cognitive Assessment (Nasreddine et al., 2005). The latter instrument, in particular, is brief, possesses reliable psychometric properties (Dalrymple-Alford et al., 2010), and successfully detects subtle deficits and MCI (Hoops et al., 2009).

Also, as Bialystok et al. (2014) maintain, broad assessments of cognitive status should be complemented with sensitive measures of executive function, as this domain is specifically modulated by bilingualism. A good option would be the INECO Frontal Screening battery (Torralva et al., 2009b), a brief tool for assessing neurodegenerative conditions (Torralva et al., 2009b; Gleichgerrcht et al., 2011), in general, and medial frontal executive functions (Roca et al., 2011), in particular. Over a maximum total score of 30 points, a 25-point cut-off score has shown a sensitivity of 96.2% and a specificity of 91.5% in detecting patients with dysexecutive syndrome (Torralva et al., 2009a). Its application may be particularly informative since CR may be modulated by executive skills both in AD and MCI (Buckner, 2004).

In brief, the relationship between bilingualism and CR may be more clearly explored by incorporating reliable measures of bilingual proficiency, lifestyle, substance use, cognitive status, and executive functioning. These would be particularly useful in studies featuring intentional samples. Moreover, they could yield crucial data to explore associations with patients' performance on experimental tasks, as described below.

### Incorporating experimental tasks

In addition to improving the toolkit used in non-experimental research, the field would greatly benefit from incorporating controlled experiments. In particular, future research should focus on domains that are sensitive to the impact of bilingualism. The literature shows that different tasks yield distinctive results for bilinguals relative to monolinguals: disadvantages in verbal processing, null effects in working memory, and advantages in other executive functions (Bialystok et al., 2009, 2012). Accordingly, if bilinguals have higher CR, these tasks should yield predictable patterns of performance when used with demented samples.

First, throughout the lifespan, bilinguals show disadvantages in single-language verbal tasks (Bialystok, 2009). This has been systematically shown through the Peabody Picture Vocabulary Test (Dunn and Dunn, 1997), in which participants must decide which of four pictures corresponds to a noun uttered by the experimenter. Experiments using this test with children and adults show that bilinguals are outperformed by monolinguals (Bialystok, 2009). Thus, if bilingualism increases CR, such disadvantages could be expected to attenuate or disappear if the task is performed by demented samples.

Second, other domains seem indifferent to the effects of bilingualism (Bialystok, 2009). For example, overall working memory performance seems to be similar in bilinguals and monolinguals. This result is particularly robust in verbal span tasks (Bialystok et al., 2008; Feng, 2009; Bonifacci et al., 2010; Namazi and Thordardottir, 2010; Engel de Abreu, 2011). Accordingly, it would be interesting to explore whether such results in non-demented samples turn into bilingual advantages when AD samples are compared.

Third, bilingualism enhances inhibitory control in non-verbal tasks. Studies using the Simon task or the Stroop task have reported better performance in bilinguals than in aged-matched monolinguals (Bialystok et al., 2004, 2008). Also, bilinguals seem to have stronger intrinsic functional connectivity in the frontoparietal control network and the default mode network, which may be beneficial in aging (Grady et al., 2015). At the same time, performance on tests of executive function decline more rapidly around 2–3 years before AD diagnosis (Grober et al., 2008). Thus, longitudinal research with AD samples could examine whether executive subdomains (e.g., inhibitory skills), relative to other domains, are less affected by disease progression in bilinguals.

Finally, tasks not widely used in the field of bilingualism may also shed light on the issue. Consider, for example, proactive interference (PI) paradigms. PI refers to the disruptive effect of prior information on retrieval of more recent information (Lustig and Hasher, 2002). That is, PI occurs when information stored in long-term memory proactively interferes with newly learned information. PI resolution involves proactive and reactive control mechanisms, which appear to be better managed by bilinguals (Morales et al., 2013a,b). PI skills are susceptible to age and cognitive decline in normal aging (Lustig et al., 2001; Bowles and Salthouse, 2003), and they may deteriorate in early stages of AD (Ebert and Anderson, 2009) and amnestic MCI (Crocco et al., 2014)—note that pre-morbid subjects may show cognitive decline several years before AD diagnosis (Amieva et al., 2005). Second, PI tasks are different from other memory measures in that items posed for recall are explicitly presented and interference effects can be assessed while controlling for initial levels of memory impairment (Crocco et al., 2014).

The earliest neuroanatomical changes in aMCI involve the hippocampus and the entorhinal cortex, two structures implicated in the integration and learning of associative information (Troyer et al., 2008). In this respect, PI paradigms may be worth considering, as aMCI may involve increased sensitivity to PI effects, independently of other associative and semantic impairment (Hanseeuw et al., 2010). Moreover, default mode network connectivity is altered in prodromal AD, including pre-MCI individuals with cognitive complaints (Sorg et al., 2007; Wang et al., 2013). Indeed, specific regions of the default network are selectively vulnerable to early amyloid deposition in AD (Sperling et al., 2010). Note that activation of the default network has been proved to enhance performance on executive control tasks when control processes engage long-term memory representation (Spreng et al., 2014), and proactive control has been associated to theta frontoparietal connectivity (Cooper et al., 2015). Thus, since proactive control is greatly taxed during bilingual processing and bilinguals haven shown to have enhanced connectivity than monolinguals in both networks (Grady et al., 2015), PI tasks can reveal specific aspects of memory variance in the onset and evolution of MCI and AD in bilinguals.

Also, increased PI demands in bilinguals may bring about neuroplastic changes that are beneficial in aging (Ansaldo et al., 2015). As this mechanism is vulnerable to brain dysfunction (Braver et al., 2007) and the PI effect has been linked to hippocampal activity in neurogenesis (Frankland et al., 2013), it may also constitute another critical source of data for the hypothesis that life-long bilingualism enhances CR. However, it is highly likely that CR relies on plastic changes other than neurogenesis, which represents a rather negligible phenomenon. More plausible candidates are dendritic sprouting, synaptogenesis, and dendritic arborization, which have been shown to occur more prolifically (Leal-Galicia et al., 2008; Gelfo et al., 2009). Moreover, CR may impact neural plasticity in AD patients by diminishing Aβ deposition (Jagust and Mormino, 2011). Besides, clinically silent pathology in normal aging suggests that CR can ameliorate brain dysfunction via plastic mechanisms even when brain pathology growths. In fact, postmortem studies of high-pathology non-demented subjects have revealed preserved density of synaptophysinlabeled presynaptic terminals and dendritic spines relative to AD patients with a similar burden of plaques and tangles (Jellinger and Attems, 2013). Although evidence for these phenomena so far comes mostly from animal models, they are probably more pervasive and relevant in humans as well. Future methodological developments may help clarify the putative neuroplastic mechanisms supporting CR in humans and, more specifically, in bilinguals.

### Further considerations

A prospective renewed framework to assess the relationship between bilingualism and CR should also contemplate additional issues. First, more homogeneous approaches should be encouraged for AD diagnosis. So far, only some studies have considered clinimetrics and cultural differences. Future studies should systematically factor in both aspects. Moreover, it would be crucial to combine behavioral paradigms with different techniques revealing possible biomarkers of AD across genetic, anatomical, and network-connectivity levels. First, note that well-established risk genes (e.g., APOE, SORL1) and causative genes (e.g., APP, PSEN1, PSEN2) for AD have been expanded to more than 20 risk loci (e.g., ABCA7, BIN1, CD33, CD2AP, CLU, CR1, EPHA1, MS4A4E/MS4A6A, PICALM; Karch et al., 2014). The pathological changes induced by genetic factors could be better understood by considering cerebrospinal fluid biomarkers (Zhang et al., 2005; Kovacs et al., 2010; Ghidoni et al., 2012; Craft et al., 2013). A challenge for the field is to explore correlations between these factors and differential neurodegenerative patterns between bilingual and monolingual AD patients.

Also, neuroimaging evidence indicates that AD is characterized by alterations in the default mode network (Sheline and Raichle, 2013), which has been found to feature stronger intrinsic functional connectivity in bilinguals than in monolinguals (Grady et al., 2015). Interestingly, proactive control has been associated to theta frontoparietal connectivity (Cooper et al., 2015) and bilinguals have proved to have better resolution of control mechanisms (Morales et al., 2013a,b). So, it would be useful to assess whether this differential pattern holds when comparing AD patients from both groups. Additionally, the research focus should move beyond detecting delays in AD symptoms onset. It would be useful to assess the impact of bilingualism in the progression of AD and associated cognitive deficits. While typical measures, such as the MMSE, are blind to these changes, experimental tasks (including PI paradigms) may reveal progressive patterns in the course of disease.

Thus, neuroimaging or electrophysiological markers could be obtained during active tasks to assist the detection progressive monitoring of cognitive changes linked to normal aging, MCI, and AD. For instance, short-term memory binding tasks (Parra et al., 2009, 2010) prove sensitive to early and even preclinical AD. This would be a particularly revealing paradigm to track the onset and progression of early AD, as short-term memory binding is not affected by normal aging (Brockmole et al., 2008; Parra et al., 2009; Brown and Brockmole, 2010; Brockmole and Logie, 2013). Moreover, PI paradigms may reveal memory variance and CR in normal aging as well as through the course of AD. PI performance decreases throughout healthy aging (Lustig et al., 2001; Bowles and Salthouse, 2003), MCI (Crocco et al., 2014), and AD (Ebert and Anderson, 2009). Thus, important insights could be gained by comparing biomarkers of these tasks between bilinguals and monolinguals with MCI or AD.

Finally, the field should expand its horizons beyond AD and assess CR in bilinguals exhibiting other disorders. In this sense, it would be interesting to explore other conditions characterized by both linguistic (e.g., primary progressive aphasia) and non-linguistic (e.g., amyotrophic lateral sclerosis, behavioral variant frontotemporal dementia) symptoms. This would pave the way for more refined insights into the possible impact of bilingualism on domain-specific CR.

## Conclusion

Research on CR in bilinguals proves very challenging because of the multiple variables involved. The limitations underlying inconsistencies across studies could be largely circumvented through experimental approaches to the issue and with more stringent control of relevant variables. Also, the field could be broadened through approaches which explore not just the delay of AD in bilinguals, but the changes occurring throughout the course of disease. These considerations could help us tease apart the potential contributions of bilingualism to preserved functioning across cognitive domains. Moreover, they may shed light not just on the relationship between bilingualism and CR, but also on more general mechanisms of cognitive compensation.

## Author contributions

All authors contributed equally to this work.

## Funding

This work was partially supported by grants from CONICET, CONICYT/FONDECYT Regular (1130920), FONCyT-PICT 2012-0412, 2012-1309, and the INECO Foundation.

### Conflict of interest statement

The authors declare that the research was conducted in the absence of any commercial or financial relationships that could be construed as a potential conflict of interest. The reviewer Stefan Elmer and handling Editor Lutz Jäncke declared their shared affiliation, and the handling Editor states that the process nevertheless met the standards of a fair and objective review.
